# 3D-microtissue derived secretome as a cell-free approach for enhanced mineralization of scaffolds in the chorioallantoic membrane model

**DOI:** 10.1038/s41598-021-84123-x

**Published:** 2021-03-08

**Authors:** Lukas Otto, Petra Wolint, Annina Bopp, Anna Woloszyk, Anton S. Becker, Andreas Boss, Roland Böni, Maurizio Calcagni, Pietro Giovanoli, Simon P. Hoerstrup, Maximilian Y. Emmert, Johanna Buschmann

**Affiliations:** 1 Division of Surgical Research, University Hospital of Zurich, Zurich, Switzerland; 2 Plastic Surgery and Hand Surgery, University Hospital of Zurich, Sternwartstrasse 14, 8091 Zurich, Switzerland; 3 Institute for Regenerative Medicine (IREM), Moussonstrasse 13, 8044 Zurich, Switzerland; 4 Hospital Limmattal, Schlieren, Switzerland; 5 Department of Orthopaedic Surgery, University of Texas Health Science Center San Antonio, San Antonio, TX USA; 6 Institute for Diagnostic and Interventional Radiology, University Hospital of Zurich, Zurich, Switzerland; 7 White House Center for Liposuction, Zurich, Switzerland; 8 Wyss Translational Center Zurich, University of Zurich & ETH Zurich, Zurich, Switzerland; 9 Department of Cardiovascular Surgery, Charité Universitätsmedizin Berlin, Berlin, Germany; 10 Department of Cardiothoracic and Vascular Surgery, German Heart Center Berlin, Berlin, Germany

**Keywords:** Biomaterials, Biomineralization, Stem cells, Adult stem cells, Mesenchymal stem cells

## Abstract

Bone regeneration is a complex process and the clinical translation of tissue engineered constructs (TECs) remains a challenge. The combination of biomaterials and mesenchymal stem cells (MSCs) may enhance the healing process through paracrine effects. Here, we investigated the influence of cell format in combination with a collagen scaffold on key factors in bone healing process, such as mineralization, cell infiltration, vascularization, and ECM production. MSCs as single cells (2D-SCs), assembled into microtissues (3D-MTs) or their corresponding secretomes were combined with a collagen scaffold and incubated on the chicken embryo chorioallantoic membrane (CAM) for 7 days. A comprehensive quantitative analysis was performed on a cellular level by histology and by microcomputed tomography (microCT). In all experimental groups, accumulation of collagen and glycosaminoglycan within the scaffold was observed over time. A pronounced cell infiltration and vascularization from the interface to the surface region of the CAM was detected. The 3D-MT secretome showed a significant mineralization of the biomaterial using microCT compared to all other conditions. Furthermore, it revealed a homogeneous distribution pattern of mineralization deposits in contrast to the cell-based scaffolds, where mineralization was only at the surface. Therefore, the secretome of MSCs assembled into 3D-MTs may represent an interesting therapeutic strategy for a next-generation bone healing concept.

## Introduction

Bone tissue engineering (BTE) has been considered as a next-generation strategy to treat large bone defects. Whereas bone has the unique ability to self-repair minor injuries^1^, large bone defects are still treated by autologous bone grafts from different origins. The iliac crest is usually preferred for this purpose, but it is associated with a variety of adverse events^2^. As a new promising strategy, tissue engineered (TE) substitutes have gained a lot of attention. Although research has made much progress in BTE over the last few decades, three major problems still remain unresolved: (1) tissue engineered grafts do not provide the regenerative potential required, thus leading to a limited effectiveness to treat bone defects^3^, (2) cell death in the core of the TEC because vessels just reach the outer shell of the scaffolds^4,5^ (3) cell retention is still limited and needs to be improved, as cells are getting lost during implantation^6^. Therefore, to achieve sufficient tissue regeneration in a TE bone graft, the selection of an appropriate cell type and format together with an appropriate biomaterial is crucial to enable in vivo performance.

Adipose tissue-derived mesenchymal stem cells (ASCs) became a popular alternative to bone marrow-derived mesenchymal stem cells (MSCs) as an autologous stem cell source for their minimal invasive accessibility by liposuction, high yields of cells and due to easy differentiation towards the osteoblast and chondrocyte phenotypes^7–9^. In an immunodeficient rat femur nonunion fractur model, ASCs have demonstrated improved therapeutic efficacy compared to human fibroblasts. Fracture healing was associated with enhanced biomechanical function and upregulation of bone morphogenetic protein-2 (BMP-2), vascular endothelial growth factor (VEGF) and angiopoietin-1 in peri-fracture tissue^10^. In addition to the preclinical stage, these stem cells were also applied in the clinical setting. In two different groups of patients with bone nonunions due to congenital pseudoarthrosis and bone tumours, respectively, ASCs were used to cure bone defects under extreme clinical and pathophysiological conditions. Autologous ASCs were supplemented with demineralised bone matrix in order to preserve the scaffold-free osteogenic 3D structure as conventional treatment was not feasible in these patients. The therapy led to restoration of bone anatomy and function with minor donor site morbidity and no oncological side effects^11^. Besides the cell-based therapy approach, the secretome of MSCs offers a cell-free alternative and contains a cocktail of many different factors, such as growth factors and cytokines. The paracrine components are responsible for the angiogenic potential, the anti-inflammatory effect and therefore also for improved bone regeneration in vivo^12–17^. Similar promising results were observed with the application of secretome in clinical trials^18,19^. This cell-free approach has the advantage of being available any time compared to the cell product itself and can consequently be used off-the-shelf.

**Figure 1:**
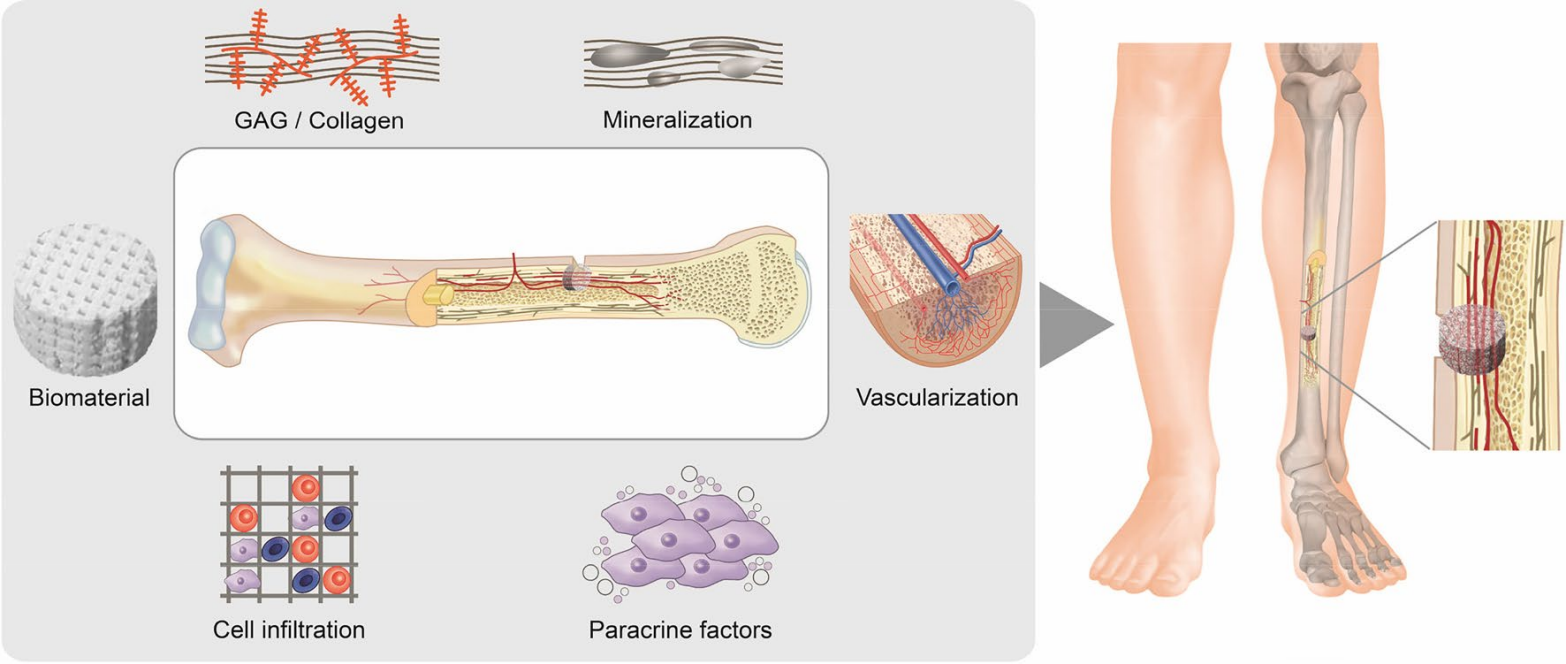
Key factors for bone regeneration in the applications of bone grafts in combination with stem cells or their secreted factors. In addition to calcification, components of the extracellular matrix such as glycosaminoglycan (GAG) and collagen play an important role in the healing process. For the functionality of the bone and the viability of the graft, the enabling of cell infiltration and thus vascularization of the construct are also important prerequisites. The selection of an appropriate biomaterial as scaffold is of fundamental relevance, as this must be able the interaction of the key factors mentioned above. When combined with stem cells or their secretomes only, the regeneration is further promoted by paracrine factors.

Recent studies have shown that, in contrast to cell type, cell format also plays an important role in the differentiation potential of stem cells, such as three-dimensional microtissues (3D-MTs), which are an aggregated format of single cells (SCs)^20,21^. They seem to have superior function compared to single cell applications because they provide an in vivo-like microenvironment to proliferate, differentiate and secret bioactive factors. Finally, they are protected from inflammatory reactions right after implantation and may therefore enhance therapeutic efficacy^22,23^. In terms of the angiogenetic potential of 3D-MTs, which is one of the key factors of cell survival and bone formation (Fig. 1), a recent in vitro study showed that that the formation of human cardiopoietic stem cells into 3D-MTs substantially increases their overall angiogenic potential and functional neovascularization capacity^24^. Furthermore, MSC-based 3D-MTs with incorporated calcium phosphate microparticles significantly promoted blood vessel reconstruction and bone formation 8 weeks after transplantation into a critical-sized defect rat model^25^. The process of mineralization of hard collagenous tissue is influenced by amorphous calcium phosphate (a-CaP), non-collagenous proteins and collagen (COL) itself^26^. The a-CaP particles act as a precursor of the thermodynamically more stable hydroxyapatite^27^, which is the most abundant inorganic mineral present in bone^28–33^. Collagen plays an active rather than passive role in the mineralization process by integrating the a-CaP into the collagen fibrils and transforming them into hydroxyapatite^34^. Collagen scaffolds are osteoinductive throughout their composition and are able to bind non-collagenous proteins, which further supports the mineralization process^35^. A recent study in a critical size bone defect in a rat model reported improved bone healing when using a titanium implant with collagen and glycosaminoglycan (GAG) coating versus a mere collagen coated plate^36^. In addition to the key factors of the extracellular matrix (ECM), we showed previously in vitro how osteogenic marker expression of human ASCs in an osteochondral interface (electrospun meshes of poly-lactic-co-glycolic acid; PLGA) depends on the amount of incorporated a-CaP nanoparticles^37^.

Hence, the chick chorioallantoic membrane (CAM) assay offers an elegant alternative with numerous advantages over rodent animal experiments such as cost effectiveness, the ease of use and its accessibility to the complete circulatory system^38^. We and others have recently shown that the CAM assay represents an efficient preclinical model to assess angiogenic potential in the context of cell therapy or biomaterials research^24,39^, but has also been used to study graft transplantation^40^. Recently, it was also implemented in BTE. Using micro-computed tomography (microCT), Moreno-Jiménez et al. showed that applying human bone cylinders from the femoral head on the CAM assay exhibited significantly more regenerated bone when compared to the control or an in vitro group^41^. The CAM model is an excellent method to study the mineralization capacity of BTE and bone allografts^41–43^. The calcium required for this process is provided by the eggshell. The allantois is located immediately below the porous shell and is involved in the mobilization of calcium for the skeletal formation of the bones of the developing embryo^44–46^. Tuan et al. found a close correlation between the differentiation of the CAM and the embryonic ossification, suggesting that the CAM is the regulatory site for calcium transport via the circulatory system^47^. Therefore, this condition offers an excellent opportunity to test biomaterials and bone grafts preclinically on an adequate blood supply for the delivery of oxygen, nutrients, progenitor cells and calcium. Only a viable and functional graft is able to promote bone healing.

In this study, we comprehensively evaluate and compare the effect of different stem cell formats (2D-SCs versus 3D-MTs) and their secretome on the mineralization capacity of a natural collagen scaffold. Considering major key factors affecting bone regeneration, a detailed evaluation was performed using the *in ovo* CAM assay model system. To provide a more in-depth understanding of the mineralization process, additional cell infiltration, neovascularization, glycosaminoglycan and collagen formation were investigated. These are relevant factors they ensure the integrity and viability of the scaffold and, through the ECM, influence bone formation and the mineralization process itself. The assessment was based on 2D-histological analysis and microCT determination to bring the mineralized scaffold into a three-dimensional context. The specific hypotheses were that (1) 3D-MTs enhance scaffold calcification and vessel ingrowth compared with 2D-single cells, (2) the secretome of 3D-MTs promotes calcification and vessel ingrowth more than the one secreted from 2D-single cells, and (3) the secretome is as potent as the corresponding cells from which it was harvested with respect to vessel ingrowth and calcification capacity.

## Results

### Time course to analyze the occurrence of key factors which influence and enhance mineralization capacity

**Figure 2:**
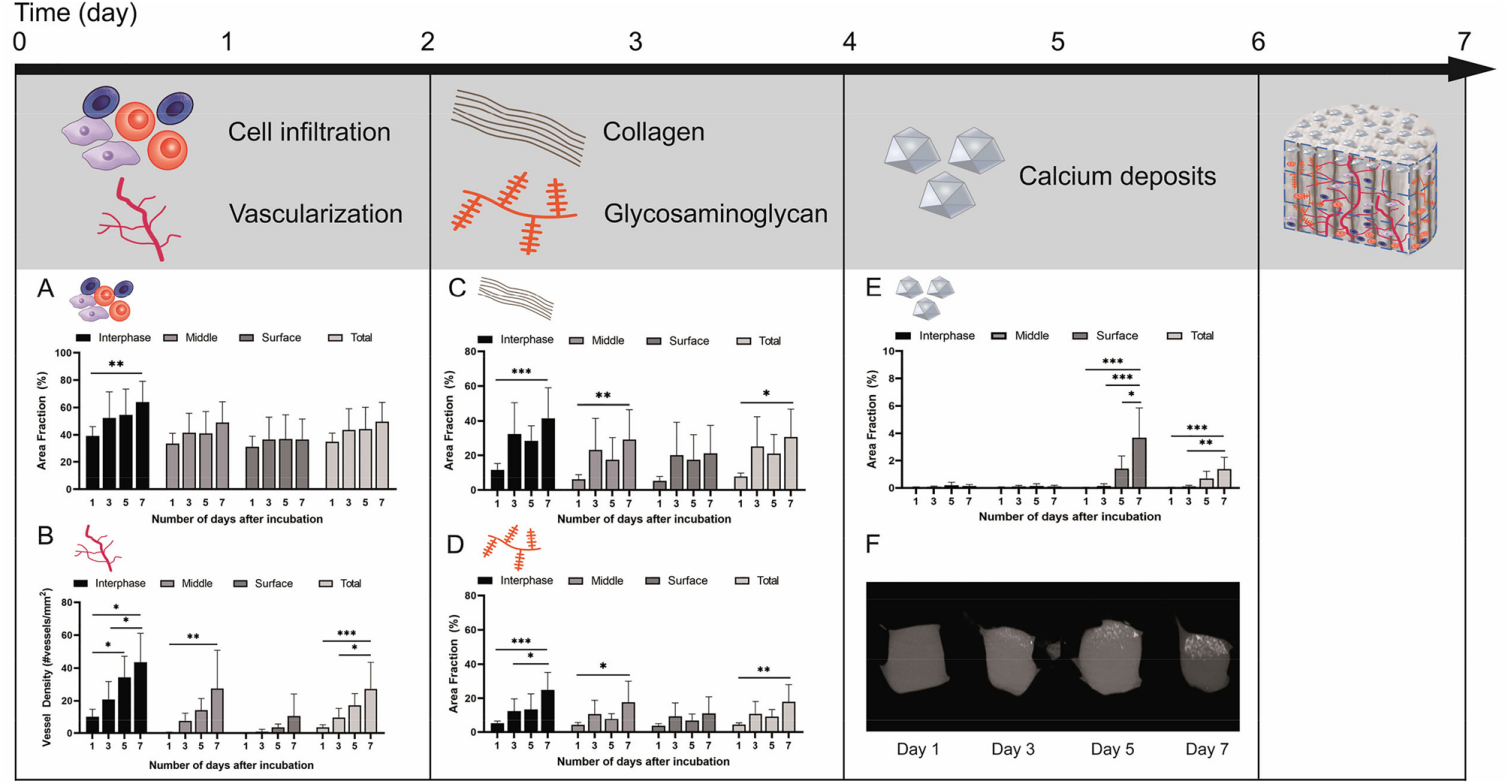
Time course regarding the occurrence and impact of key factors on the calcification process analyzed in collagen scaffold. The timing of the various factors involved was investigated (**A**–**F**) using the CAM assay. A marked increase in cell infiltration and vessel density in the scaffold was observed on day 1 (**A**, **B**). On day 3, there was a distinct increase in collagen and glycosaminoglycan (GAG) content (**C**, **D**) and from day 5 on, an accumulation of calcium deposits could be sufficiently detected (**E**), which could also be shown by microCT analysis (**F**). Schematic scaffold with the localization of key factors is shown after 7 days of incubation on the CAM.

The feasibility and potential of mineralization of a collagen scaffold *in ovo* was investigated by a time course experiment. The scaffolds were soaked with physiological phosphate buffered saline (PBS) solution and incubated for 1 week on the CAM. On day 1, 3, 5 and 7 of incubation, a histological analysis was performed. In order to obtain a more precise indication in which zone of the scaffold the initial processes occur and how they can be characterized throughout the entire scaffold area, the scaffold was divided into the three regions, i.e. interface, middle and surface. Our analysis showed that cell infiltration (Fig. 2A) and blood vessel ingrowth (Fig. 2B) started instantly after incubation. On day 1 a cell infiltration area fraction of 30.19 ± 6.84% (Mean ± SE) and a vessel density of 10.23 ± 4.57 vessels/mm^2^ were detected and had spread over 7 days towards the surface region with 36.60 ± 14.99% and 10.70 ± 13.46 vessels/mm^2^. Within a week of incubation, the infiltration rate and vessel density were increased significantly in the interface region to a maximum amount of 63 ± 15.39% and 43.54 ± 17.63 vessels/mm^2^ and respectively (Fig. 2A,B).

After 3 days of incubation a distinct increase in the collagen (Fig. 2C) and glycosaminoglycan (Fig. 2D) formation was observed, predominantly in the interface region (43.38 ± 17.90% for COL; 5.22 ± 7.13% for GAG) with a decreasing content towards the surface region (30.40 ± 19.18% for COL; 3.74 ± 7.86% for GAG). Once more comparing day 1 and day 7 with each other in both different tissues analyzed, the interface, middle and total area had shown a significance difference over time. The mineralization process (Fig. 2E,F) had increased significantly after day 5 and was mainly detected in the surface region. On day 7, the amount of total mineralization was significantly higher than over all previously measured time points within the respective region.

Taken together the time course illustrated the sequence in which the individual key factors develop (Supplementary Fig. S1). Mineralization was present in the surface region from day 5 onwards. In contrast, cell infiltration and blood vessel ingrowth in the interface region began instantly after incubation on the CAM, followed by collagen and GAG formation on day 3.

### Influence of stem cell format on cell infiltration, cell density and vascularization of collagen scaffold

**Figure 3:**
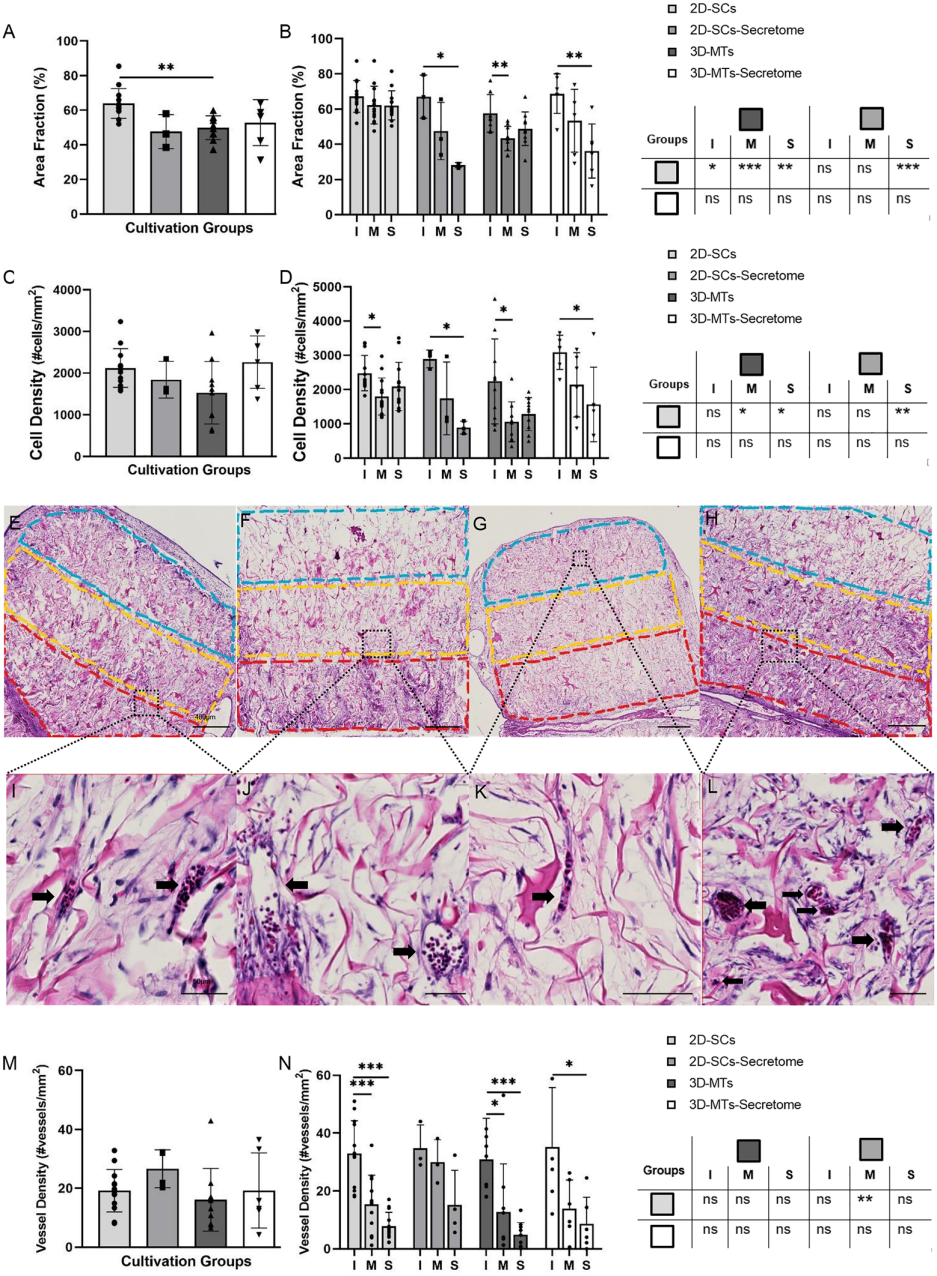
Histological analysis of cell infiltration (%) and cell density (cells/mm^2^) into the natural collagen scaffold (Optimaix). Histological analysis of the infiltration (**A**, **B**), cell density (**C**, **D**) and vessel density (**M**, **N**) of the four cultivation groups are assessed on day 7. Results are presented in a group comparison of the total scaffold (**A**, **C**, **M**) and more specified in differing three ROIs (interface (I = red), middle (M = yellow) and the surface (S = blue)) shown in (**B**, **D**, **N**). Significances of intra-group calculations are demonstrated within the graph whereas inter-group differences with its corresponding ROIs are listed on the separate table next to the graph (**B**, **D**, **N**). The results are illustrated with an exemplary out-take of each experimental group using single cells (**E**, **I**), 2D-SCs secretome (**F**, **J**), 3D-microtissues (**G**, **K**), 3D-MT secretome (**H**, **L**). The scale bar was defined with 400 µm in the overview examples (**E**–**H**) and 50 µm for the more detailed examples (**I**–**L**). Statistical results were established with a one-way ANOVA considering only one independent factor and a two-way ANOVA considering two categorical factors and the effect of the categorical factors on each other. P-values were considered significant by the APA-System: 0.12 (ns), 0.033 (*), 0.002 (**), < 0.001 (***).

Corresponding to the time course, cell and tissue infiltration as well as the formation on neo-vessels, starting already at day 1 after incubation from the interface region and spreading up to the surface region over time, the influence of the different stem cell formats, 2D-SCs vs 3D-MTs, and their corresponding secretome was analysed (Fig. 3). At day 7, cross sections were stained with haematoxylin and eosin staining (H&E; Fig. 3E–L) and the area fraction (%) as well as cell and vessel densities were assessed.

Focusing on differences across the entire scaffold between the four experimental groups with respect to tissue infiltration (Fig. 3A), only 2D-SCs group (63.90 ± 8.65%) showed improvement a higher infiltration rate compared to the 3D-MTs (49.88 ± 6.87%). The cell density (Fig. 3C) did not differ significantly between the groups.

Within one scaffold between the three different region of interests (ROIs), the interface region of was mostly affected in terms of cell infiltration (Fig. 3B,E–H) and cell density (Fig. 3C,E–H) with a decline in their content to the upper regions. Exceptionally therefore was the infiltration capacity of 2D-SCs (Fig. 3E), which interestingly showed a homogenous distribution over the entire scaffold (interface: 67.25 ± 9.04%, middle: 62.32 ± 10.76%, surface: 62.14 ± 8.19%). A similar pattern was also found in the 3D-MTs group, but with significantly less pronounced area fractions. This homogenous distribution in the 2D-SCs group with high contents of tissue infiltration up to the top of the scaffold led in the inter-group comparison (Fig. 3B) to superiority of the surface region of 2D-SCs (62.14 ± 8.19%) compared to its secretome (36.20 ± 15.31%) and to the 3D-MTs (48.85 ± 9.51%). Similar findings were made regarding cell density (Fig. 3D) where in the middle region 2D-SCs (1797.64 ± 540.11 cells/mm^2^) were superior compared to 3D-MTs (1057.42 ± 582.30 cells/mm^2^) and in the surface region (2090.76 ± 701.01 cells/mm^2^) had an advantage compared to the 3D-MTs (1285.61 ± 479.39 cells/mm^2^) and its secretome group (1564.66 ± 1088.91 cells/mm^2^).

Comparing vessel density of the entire scaffold (Fig. 3M) between all four groups, highest numbers were counted in the 2D-secretome group (26.64 ± 6.42 vessels/mm^2^) by trend. The highest neo-vessel development was found in the interface to the connecting CAM (Fig. 3N), with values between 30 and 40 vessels/mm^2^. Vascularization into the middle region was interestingly most prominent in the 2D-SCs secretome group (29.99 ± 7.72 vessels/mm^2^) and was even better than found for cell-seeded 2D-SCs.

Briefly, our data suggest that tissue infiltration and cell density go along closely, and the interface was the predominantly affected region with highest contents in the 2D-SC group. In terms of vascularisation, however, the 2D-SC secretome group was able to induce the most vessels up to the middle region and was superior compared to its cell-seeded counterpart.

### Collagen and glycosaminoglycan formation

**Figure 4:**
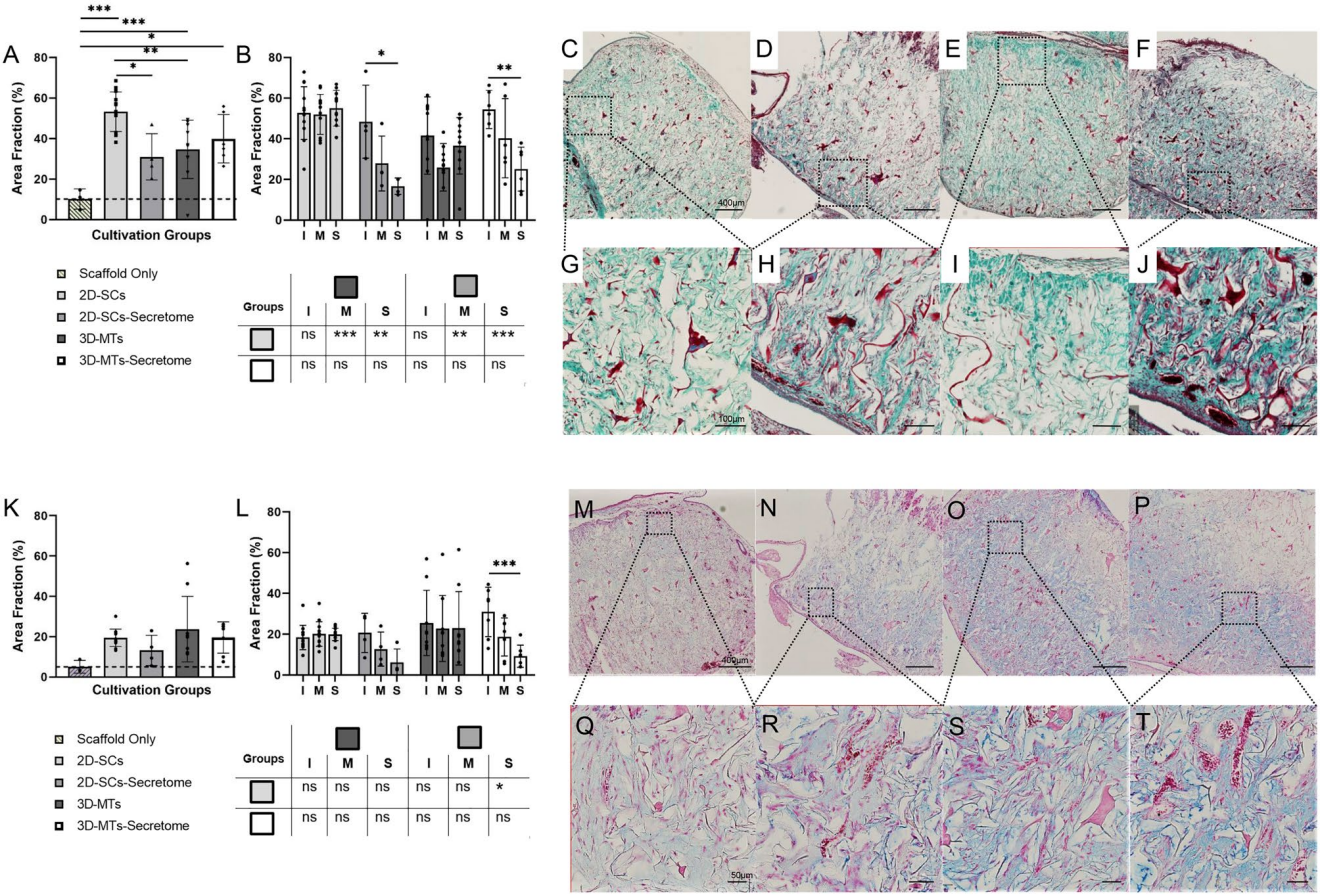
Histological analysis of collagen and glycosaminoglycan formation into the natural collagen scaffold (Optimaix). Histological analysis of collagen (**A**–**J**) and GAG (**K**–**T**) of the four cultivation groups are assessed on day 7. An additional scaffold only group was assessed to provide a reference what amount is stained by the scaffold itself. Results are presented in a group comparison of the total scaffold (**A**, **K**) and more specified in differing three ROIs (interface (I), middle (M) and the surface (S)) (**B**, **L**). Significances of intra-group calculations are demonstrated within the graph whereas inter-group differences with its corresponding ROIs are shown on the separate tables. The results are illustrated with an exemplary out-take of each cultivation group using single-cells (**C**, **G**, **M**, **Q**), 2D-SCs-secretome (**D**, **H**, **N**, **R**), 3D-microtissues (**E**, **I**, **O**, **S**), 3D-MT-Secretome (**F**, **J**, **P**, **T**). An exemplary part of the interface region is shown for each group (**G**–**J**, **Q**–**T**). The scale bar was defined with 400 µm in the overview examples (**C**–**F**, **M**–**P**) in both stains and 100 µm for the more detailed examples of collagen formation (**G**–**J**) and 50 µm of GAG formation (**Q**–**T**). Statistical results were established with a one-way ANOVA considering only one independent factor and a two-way ANOVA considering two categorical factors and the effect of the categorical factors on each other. P-values were considered significant by the APA-System: 0.12 (ns), 0.033 (*), 0.002 (**), < 0.001 (***).

It is well known that collagen and glycosaminoglycan play an active role in bone formation^34–36^. Therefore, we had histologically analysed a collagen scaffold with either a cell-based cultivation using 2D-SCs and 3-MTs or a cell-free cultivation using their corresponding secretome regarding COL-formation with a Masson-Goldner-Trichrome stain or GAG-formation with an Alcian Blue stain respectively. As a reference to what amount of positive staining is caused by the fibers of the scaffold itself, a cell-free version of the scaffold was cultured and analyzed (Fig. 4).

Starting with the five different experimental groups and their effect over the entire scaffold (Fig. 4A,K), it appeared that every group except the 2D-SCs secretome group was significantly more positively stained as the reference group (10.14 ± 5.00%). The 2D-SCs-based scaffold contained significantly more collagen (53.22 ± 9.79%) than its corresponding secretome (30.96 ± 11.38%) and 3D-MTs (34.67 ± 14.35%; Fig. 4A). However, GAG production was thereby not affected (Fig. 4K). Although no significant changes in GAG production could be detected in relation to the overall scaffold, a detailed analysis was performed, dividing the scaffold into the three regions of interest, the interface, the middle and the surface. These regions were compared within the same group (Fig. 4B,L) for COL and GAG, respectively. In general, cell-seeded approaches had a COL and GAG content that was more homogenous (Fig. 4C,E,M,O), whereas their secretome counterparts presented a stepwise decline towards the surface (Fig. 4D,F,N,P). This finding led to significant more COL formation in the interface compared to their surface region of the 2D-SCs secretome (48.42 ± 17.97% vs. 16.67 ± 4.03%) and the 3D-MTs secretome group (54.41 ± 9.40% vs. 25.08 ± 10.82%; Fig. 4B). Same observation was made in the 3D-MT secretome group (30.93 ± 12.11% vs. 9.24 ± 5.49%) for the GAG formation (Fig. 4L).

The fact that COL has its highest yields in the 2D-SCs experimental group (middle: 51.97 ± 9.88%, surface: 55.03 ± 8.82%) was confirmed by significant differences compared to the middle region and surface region of their corresponding secretome and the 3D-MT group (Fig. 4B). Similar findings were made when compared the surface region of GAG formation between 2D-SCs (19.90 ± 7.38%) and their cell-free approach (6.61 ± 6.58%; Fig. 4L).

In summary, our results show that the distribution of both collagen and glycosaminoglycan demonstrated a quite similar and homogenous distribution in 2D-SC und 3D-MT groups, while the cell-free approaches were associated with a decrease in content up to the surface region. Highest ECM contents were produced of the 2D-SC group regarding COL formation and less pronounced in GAG formation.

### Mineralization potential of a collagen scaffold combined with stem cells or its secretome

**Figure 5:**
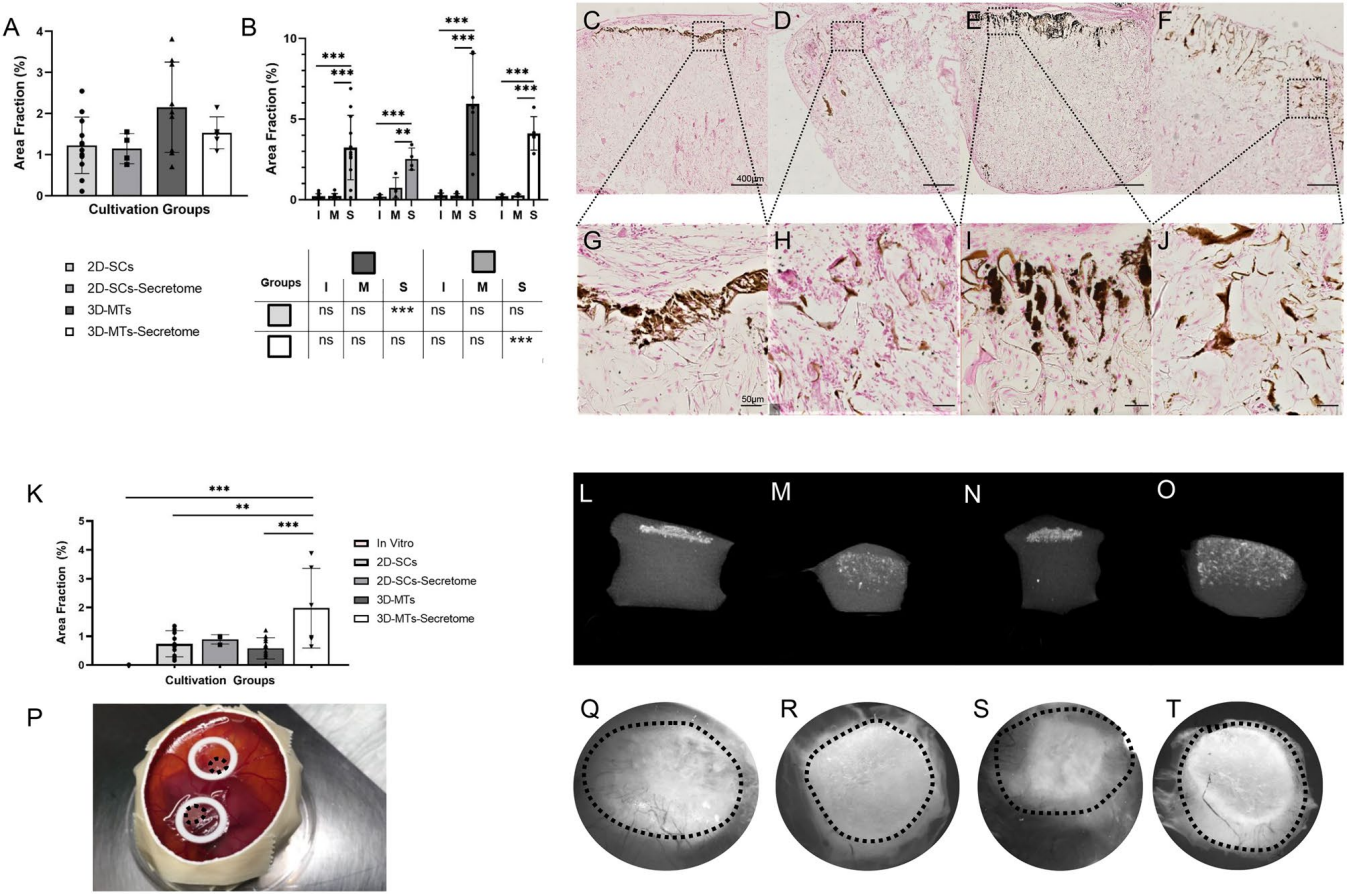
Histological and microCT analysis of the calcification potential of ASCs in the natural collagen scaffold (Optimaix). Calcification was assessed with Von Kossa histological analysis (**A**–**J**) and a microCT analysis (**K**–**O**). Results are presented in a group comparison of the total scaffold (**A**, **K**) and more specified in differing three ROIs (interface (I), middle (M) and the surface (S); shown in **B**). Significance of intra-group calculations are demonstrated within the graph whereas inter-group differences with its corresponding ROIs are shown on the separate table, bellow the graph (**B**). The results are illustrated with an exemplary out-take of each cultivation group using single-cells (**C**, **G**, **L**, **Q**), 2D-SCs secretome (**D**, **H**, **M**, **R**), 3D-microtissues (**E**, **I**, **N**, **S**), 3D-MT secretome (**F**, **J**, **O**, **T**). An exemplary part of the surface region is shown for each group (**G**–**J**). Two scaffolds were placed as onplants on the CAM of each chicken egg (**P**). View of the scaffolds from the top shows a tendency of calcification (**Q**–**T**). Dotted line marks edge of scaffolds. The scale bar was defined with 400 µm in the overview examples (**C**–**F**) and 50 µm for the more detailed examples (**G**–**J**). Statistical results were established with a one-way ANOVA considering only one independent factor and a two-way ANOVA considering two categorical factors and the effect of the categorical factors on each other. P-values were considered significant by the APA-System: 0.12 (ns), 0.033 (*), 0.002 (**), < 0.001 (***).

Mineralization capacity was evaluated with two different quantitative methods. Firstly, calcium phosphate was histologically detected with Von Kossa staining (Fig. 5A–J) which was followed by the assessment of the mineralized area fraction of total scaffold determined by microCT analysis (Fig. 5K–O).

A two-dimensional histological analysis showed that the mineralization potential did not differ significantly between the four experimental groups with respect to the overall scaffold (Fig. 5A). The 3D-MTs group hat the highest amount of mineralization deposits (5.93 ± 3.14%) and there was a trend in favour of both 3D-MT groups. Splitting the scaffolds into three ROIs (Fig. 5B), the surface region was mostly mineralized in all experimental groups compared to the other regions. Furthermore, the 3D-MTs showed the highest yields in the surface region, even significantly higher compared to the 2D-SCs. The comparison of their corresponding secretome with each other had shown comparable effects. Specifically, the 3D-MT secretome showed significantly more detectable mineralization deposits than its 2D-SC counterpart (4.11 ± 1.04% vs. 2.52 ± 0.68%).

Interestingly, the 3D-MT secretome group (1.98 ± 0.38%) was superior to the all other experimental groups in case of microCT analysis (Fig. 5K–O) with respect to mineralization deposits. The other remaining groups such as 2D-SC (0.56 ± 0.44%), the 3D-MT (0.58 ± 0.37%) and the 2D-SC secretome (0.89 ± 0.19%) therefore showed lower values. A 7 day in vitro cultivation of the scaffold with 2D-SCs showed no signs of mineralization, neither by histological analysis nor by microCT (Fig. 5K). Interestingly, mineralization in cell-based cultures (Fig. 5L,N) were mainly observed in the surface area, where secretome-based cultures mineralized more diffusely throughout the scaffold (Fig. 5M,O).

After the incubation on the CAM (Fig. 5P), the surface of the collagen scaffolds were additionally examined under the microscope. It was found that on day 7 the mineralization extent could already be estimated in the different regions without quantitative analysis (Fig. 5Q–T).

Taken together, in a 2D-histological analysis mineralization was found the most in the 3D-MT group predominantly in the surface region and was superior to the 2D-SC group. Analysing the scaffold using microCT showed more diffuse enhancement in the 3D-MT secretome group than the cell-seeded counterpart and stood out when using a three-dimensional analysis method.

## Discussion

Bone tissue engineering still faces major challenges, such as donor site morbidity, limited availability and poor vascularization. The ultimate goal is to deliver a viable and qualitative high-end bone substitutes for the treatment of large bone defects which may overcome the standard of care^48^. To promote the regeneration process, stem cells may have a beneficial impact by secreting bioactive molecules (paracrine effects). Hence, next-generation therapeutic concepts should combine state-of-the-art biomaterials and stem cells with a particular focus on the appropriate format.

**Figure 6:**
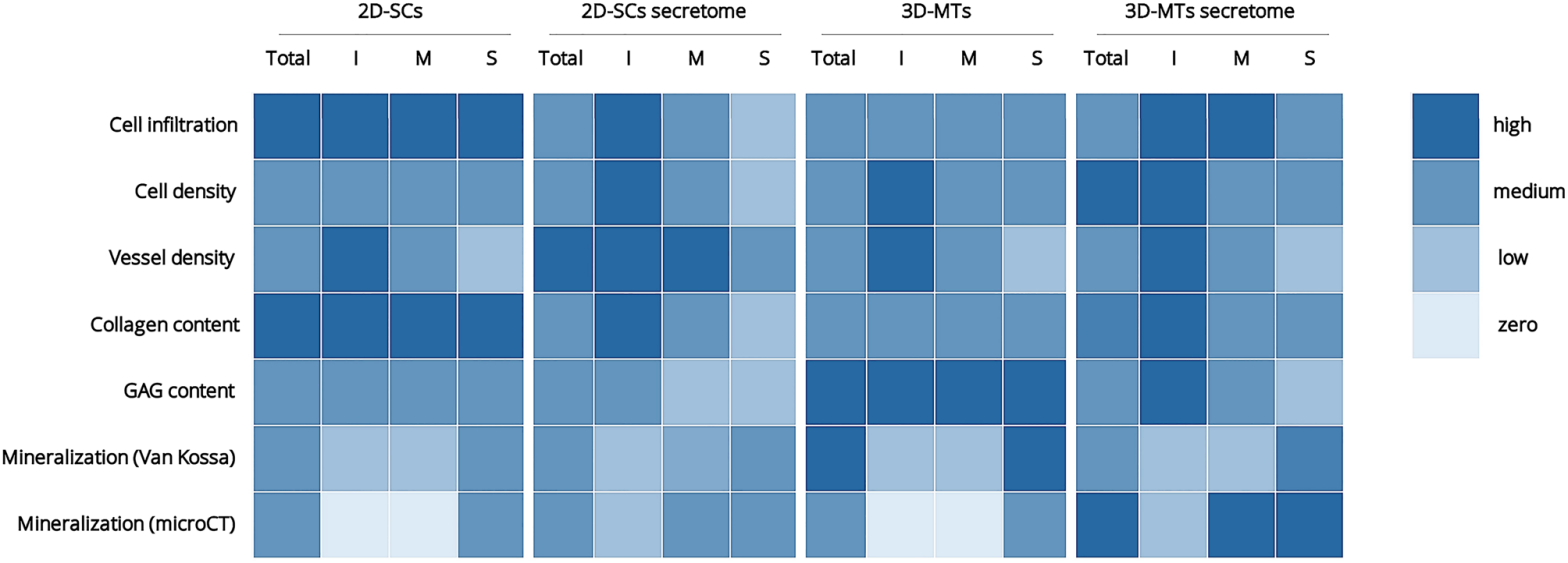
**Heat map showing overview of key results**. All quantitative results were assigned to one of the following categories zero, low, medium and high and color-coded accordingly. The investigated scaffolds of the four experimental groups 2D-single cells (2D-SCs), 3D-microtissues (3D-MTs) and corresponding secretomes were either analyzed as an entire scaffold (Total) or one of the three regions interface (I), middle (M) and surface (S).

**Figure 7:**
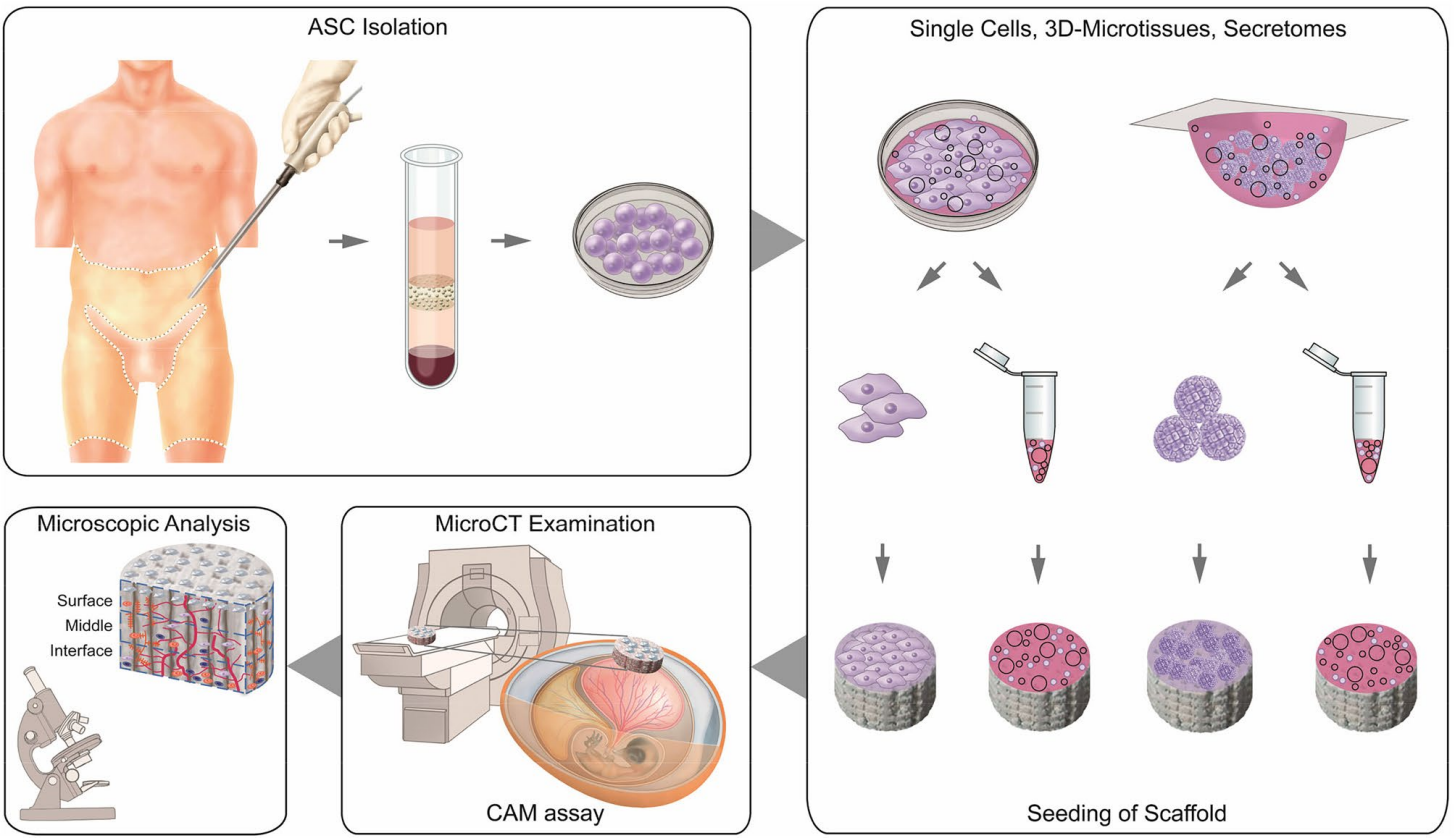
**Schematic experimental setup for the investigation of calcification capacity of Optimaix scaffold in combination with human adipose tissue-derived mesenchymal stem cells or secretome**. After isolation of human ASCs, the cells are cultured either as 2D -single cells or as 3D-microtissues. Afterwards, they are seeded onto collagen scaffolds. The secretomes of both cell formats are also added to separate scaffolds. After incubation of the scaffolds on the CAM, microCT analysis is performed to determine the degree of calcification and followed by a comprehensive histological assessment.

In our study, we used a scaffold-based approach and human ASCs to investigate the influence of 3D-microtissues in comparison to common 2D-single cells and their corresponding secretomes, on several key factors regarding the osteoconductive potential in bone formation (Fig. 6)^49^. A commercially available porcine collagen scaffold, Optimaix^24,39^, already known in bone regeneration^50^, was used as a biomaterial. It was combined with the different approaches (Fig. 7) and applied in the CAM assay. Afterwards histological analysis with respect to cell infiltration, vascularization potential, collagen and glycosaminoglycan formation and mineralization potential of the combined biomaterial stem cell bone graft was performed. In parallel, microCT analysis was used as a second objective method to quantify mineralization of the scaffolds. Following the chronological sequence shown in Fig. 2, the different changes in the scaffold over the 7 days of incubation are discussed in detail with regard to bone forming key factors (Fig. 6).

At day 1 of incubation on the CAM, tissue infiltration and neovascularization started to develop from the interface region to the surface (Fig. 2A,B). A fast tissue infiltration and high cell densities are the key factors for integration of the scaffold into the transplanted site^51,52^. Modulation of this effect can be achieved by varying the pore size^53–55^, the stiffness^56–58^ or the surface composition^59^. Even if pore sizes between 100 and 150 μm are preferred in terms of bone formation, smaller pores as found in our scaffold still can build osteoid^54^. Considering the use of biodegradable material suggests that this osteoid will transform into bony tissue while the whole collagen scaffold is resorbed. Results in this study demonstrated that 2D-SCs promoted higher infiltration throughout the chosen collagen scaffold than 3D-MTs. Infiltrated cells could consist of mesenchymal origin, such as fibroblasts^24^.

Furthermore, sufficient vascularization remains a major problem, affecting the viability of the scaffold in the long term. Every tissue with a greater diameter than 100 μm limits the range of oxygen and nutrition supply to mere diffusion and depends on a newly formed vascular system if central core necrosis wants to be prevented^4,60^. The importance of a highly functional vascular system is not only necessary for the viability of the scaffold itself, but also to enhance osteogenesis directly^61,62^. Therefore, VEGF plays a double role, both in promoting endothelial cell migration and proliferation and in stimulating osteogenesis by the regulation of osteogenic growth factors^63^. It is known that microtissues can enhance VEGF production size-dependently by inducing hypoxia-inducible factor 1α (HIF-1α) by its hypoxic core^64^. In our study, vessel density was highest only by trend in the 2D-SCs secretome group over the whole scaffold (Fig. 3M). More detailed analysis revealed that neovascularization mostly took place from the interface region with a decline in vessel density towards the surface region (Fig. 3N). Vessels in the 2D-SC secretome group, however, were newly formed predominantly in the middle region and were significantly different compared to 2D-SCs only. Considering these findings, its secretome was just as potent as the cell-based cultivation itself. These can be explained by the paracrine factors influencing neovascularization to a high degree^65,66^.

Glycosaminoglycans (GAGs) have become a powerful tool in TE the last years and drew attention for the creation of new biomaterials^67–69^. They provide biocompatible, biodegradable and non-immunogenic properties that have made GAGs useful in clinical applications^70^. Hyaluronic acid (HA), the simplest GAG and one of the major components in the ECM, affects proliferation, differentiation and cell migration under in vitro conditions^71–76^. Many studies have shown significant improvement in bone formation by applying HA to different filler materials, such as octacalcium phosphate granules, MegaGen synthetic bone or carbon nanotubes—even without the use of growth factors^77,78^. De Brito Bezerra et al. for example applied a HA gel (1%) in combination with an absorbable collagen sponge in a 5 mm critical size defect in rats and demonstrated that bone healing with HA was superior to the collagen sponge only^79^.

Regarding GAG formation over the entire scaffold, no significant difference between the four experimental groups could be distinguished in our study (Fig. 4K). In contrast to our expectation, the cell-based groups induced more homogenous GAG production with relatively equal contents in each zone, whereas both secretome groups showed a constant decline from the interface to the surface region. Against the assumption that secretome might diffuse more easily through the scaffold and therefore evoke GAG production evenly up to the surface, we found this effect only for the cell-based groups. However, a comparison to the results of tissue infiltration and cell density supports the findings for GAGs because similar trends were found. In other words, only where enough tissue infiltration with a high enough cellular density is provided ECM components can be formed. In addition, the stem cells secrete paracrine factors in a continuous way from the surface and thus can stimulate infiltration and ECM formation equally in all regions.

Collagen has also major relevance in the formation of new bone, by integration of amorphous calcium phosphate into the fibrils and transforming it into hydroxyapatite^34,35^. Moreover, osteoblastic cells produce a complex ECM which is composed of proteoglycans, collagens and non-collagens. The interplay between proteoglycans with matrix effector molecules such as the chains of GAGs or their core protein is essential for regulating a variety of cellular processes^75^. Collagen formation was supported particularly by 2D-SCs in our study, with significantly higher yields than found for its corresponding secretome or the 3D-MT groups (Fig. 4A). Cell-based experimental groups showed a more homogenous COL production than their secretomes, like found for GAGs (Fig. 4F).

Many studies have shown an excellent vascularization potential and efficiency of scaffolds using the CAM assay, which suggests to use it as an convenient model to test biomaterials and bone allografts^80,81^. However, there are only a few studies regarding their calcification potential^41–43^. In our comparison of whether bone formation is enhanced in one of the two cell formats, we were able to demonstrate that in a histological analysis 3D-MTs showed more mineralization. This either by a trend regarding the entire scaffold or by a significance mainly in the surface region where the mineralization phases were always primarily deposited. In a previous study, similar results were obtained by seeding ASC-based 3D-MTs and 2D-SCs on electrospun nanocomposites in vitro. Under static conditions, osteogenic and angiogenic markers increased in the 3D-MT group compared to the 2D-SC group^82^. Similar findings were made when ASCs where incorporated in a mineralized spheroid composite and were cultured in vitro without osteogenic supplement medium. Twenty times higher RUNX2 gene expression was found compared to the no mineral fiber group^83^. The question arises whether the stem cells themselves or their secretome alone should be used in combination with the collagen scaffold to best support mineralization. The cell-based cultures are more influenced by their autocrine factors^84^ mineralizing their ultimate vicinity, while their corresponding secretome diffuses more into the scaffold acting via paracrine factors and probably influencing stem cells originated from the chicken embryos.

There were no significant differences in our study between the cell-based groups and their corresponding secretome (Fig. 5A,F), indicating that there was no superiority. In contrast, the microCT results (Fig. 5K–O) showed a clear difference in the mineralization pattern within the scaffold and between the experimental groups. Both secretome groups showed a more diffuse and homogeneous mineralization throughout the scaffold and were increasingly detectable not only in the surface region. The area fraction of mineralization deposits in the 3D-MT secretome group was clearly superior to all other groups. We conclude that the 3D-MT secretome is the favorite regarding an optimum mineralization here.

In future studies, we need to investigate the effect and the composition of the 3D-MT secretome in more depth (proteomics) and propose it as convenient alternative to cell-based treatment. The advantage of a “off-the shelf” secretome is its readiness and long-term preservation—two aspects that might otherwise pose problems in cell therapies. The next step is the implementation into a rodent in vivo model to perform a long-term study of the tested biomaterial combined with 3D-MT secretome in a bone defect. Here, the regeneration and remodeling processes are expected to be more pronounced due to additional mechanical and biochemical stimulus^85–87^. In addition, it will allow to further investigate the paracrine effects of the 3D-MT secretome in relation to the bone healing process.

Our study has a several limitations: first, the CAM assay is restricted to 7 days, which limits the time available for the examination. Second, mineralization deposits were detected with a Von Kossa stain which did not differ in which phase calcium is present on a molecular level^88^. Third, histological slides allow analyses in a two-dimensional manner and only allow extrapolations to be made about the entire scaffold. Fourth, in order to characterize the infiltration of individual cell types and to perform further comprehensive histological analyses, addressing differentiation processes of stem cells and the presence of osteogenic cells, specific antibodies are rare if not unavailable for the chicken species. Finally, whenever possible, PCR technique may be used for further characterization and quantification of specific markers, as far as chicken-specific primer sequences are known and available.

In summary, we here evaluated a biodegradable collagen-based scaffold which was either seeded with human MSCs as single cells or as 3D-microtissues and compared to the scaffold soaked with the corresponding secretomes. The results confirmed in all experimental groups an accumulation of collagen and GAG in the scaffold over time, as well as a pronounced cell infiltration and vascularization. All conditions showed an osteoconductive potential, whereas the soaking with secretome of 3D-microtissues was superior in regard to mineralization capacity showing a more homogeneous distribution of mineralization deposits within the scaffold and it also promoted cell infiltration from host tissue. In addition, it is simple to preserve, and its instant readiness makes it a good off-the-shelf candidate. Therefore, the secretome of MSCs combined with a collagen scaffold may be considered as an interesting approach for a cell-free next-generation functional bone graft.

## Methods

### Study design

Human mesenchymal stem cells were isolated from lipoaspirate material. The cells were then cultivated either as single cells or in hanging drops as 3D-microtissues. After 3 days, both the cells and their corresponding secretome were harvested. Collagen-based Optimaix scaffolds were seeded with either 2D-SCs, 3D-MTs or corresponding secretomes and incubated on the CAM for 7 days. For the detection and quantification of mineralization deposits, the scaffolds were examined by microCT and then processed further for histological analysis. A comprehensive quantitative analysis of all relevant key factors (Fig. 1) was thus achieved (Fig. 7). All experimental protocols were approved by the Canton Zurich ethical committee (KEK-ZH-Nr. 2010-0476/0), Switzerland. Informed consent of patients was obtained for the isolation of ASCs from lipoaspirate.

### Cell culture

Human ASCs (n = 3) were isolated from lipoaspirate after obtaining the written patient’s consent. All protocols were conducted in accordance to the Cantonal Ethics Committee Zurich in Switzerland (KEK-ZH-Nr. 2010-0476/0). Cells were processed and cultivated as described elsewhere^89^. Human ASC-based 3D-MTs were generated using the hanging drop method^90^. For this purpose, 10,000 cells/ml were seeded into Terasaki microtest plates (Greiner Bio-one, Germany). Drop sizes with a volume of 25 μl were incubated upside down under standard conditions for 3 days. Both the harvested 3D-MTs and the 2D-SCs were washed with phosphate buffered saline (PBS; Sigma Aldrich, Switzerland) and resuspended in 30 μl serum-free medium (SFM) for seeding onto the scaffold. To prepare the conditioned medium, 2D-SCs as well as 3D-MTs were cultivated in a concentration of 10,000 cells/ml in SFM (DMEM high glucose, Sigma Aldrich, Switzerland) for 3 days. The supernatant was then harvested as secretome and followed by removal of cell components using a centrifugation step.

### Scaffolds

Optimaix (Matricel GmbH, Herzogenrath, Germany) is a porcine collagen sponge, which is biodegradable in vivo. The scaffolds had a size of 5 mm in diameter and 3 mm in height and contained highly oriented pores of 80–100 μm. This guiding structure allows the infiltration of host cells and newly formed blood vessels.

For cell seeding, either 500,000 cells were applied as 2D-SCs or 3D-MTs in 30 μl SFM per side of scaffold or the scaffold was soaked with 30 μl secretome per side (top and bottom). For the time course experiment the scaffolds were soaked with 30 μl PBS per side.

### CAM assay

The CAM assay was performed as described elsewhere^24^, using fertilized Lohmann white LSL chick eggs (Animalco AG Geflügelzucht, Switzerland). No IACUC approval is required until embryonic day 14 according to Swiss animal care guidelines (TSchV, Art. 112). Briefly, on incubation day (ID) 3.5 a windowing of the eggshell was carried out. All incubation steps were performed at 37 °C and 65% relative humidity. On ID 7, Optimaix scaffolds seeded with either 3D-MTs, 2D-SCs or with the respective secretomes, were carefully placed on top of the CAM, two scaffolds onto each egg (Fig. 5P). The eggs were then incubated for another 7 days. For the time course study the eggs were incubated until ID 8, 10, 12 and 14.

### Microcomputed tomography and analysis

The scaffolds fixed with 4% phosphate-buffered formalin solution (Formafix, Switzerland) were scanned using microCT Skyscan 1176 device (Bruker BioSpin AG, Fällanden ZH, Switzerland). The scanning conditions were as follows: isotropic nominal resolution of 18 µm, 50 kV, 500 µA, exposure of 280 ms, and two-fold frame averaging. All data was processed using manufacturer’s software and ImageJ (version 1.50i) software. The images were changed into an 8-bit format and then a Z projection was made. Thresholds were determined to a value, which suppressed all the background activity.

### Histology and analysis

Paraffin embedded and with 4% phosphate-buffered formalin solution fixed scaffolds (between n = 3 and n = 13 per group) were sagitally bisected and from there a 5 μm section with the three zones (interface, middle, surface) was examined in more detail. A staining with Hematoxylin (Artechemis, Switzerland) and Eosin (Waldeck, Germany; H&E) was performed to quantify cell infiltration, cell density and vessel density. To verify the mineralization and ECM contents of the scaffold, a staining with Von Kossa (5% silver nitrate solution (Roth, Switzerland), 5% sodium thiosulfate (Morphisto, Germany), nuclear red (Morphisto, Germany)), respectively Alcian blue (3% acetic acid (Sigma Aldrich, Switzerland), alcian blue (Morphisto, Germany), 0.1% nuclear fast red (Morphisto, Switzerland)) and trichrome masson blue (ponceau fuchsin (Roth, Switzerland), tungstophosphoric acid orange G solution (Roth, Switzerland), light green in acetic acid (Roth, Switzerland)) was carried out. Images were taken with a digital slide-scanner NanoZoomer using NDP.view2 software (Hamamatsu Photonics, Japan). The scans were made with a 40× zoom which is corresponding to a pixel resolution of 0.230 µm. The quantitative evaluation was performed for the entire scaffold section and for the three zones interface, middle and surface (Fig. 7). Area fractions of the positive staining were determined using Image J Fiji follow the example of the concept of Chen et al.^91^. The channels of the plug in “Color Deconvolution” were chosen dependent on the corresponding stain. The threshold for each stain was selectively chosen to suppress the background activity. The area fraction was determined as the quotient of the positive staining compared to the whole area in percent. The cell density and vessel density were determined using QuPath software^92^. The numbers of counted cells and vessels were standardized to the area of one mm^2^.

### Statistics

Statistical analysis was performed using GraphPad Prism 8. Results were established with a one-way ANOVA considering only one independent factor and a two-way ANOVA considering two categorical factors and the effect of the categorical factors on each other. P-values were considered significant by the APA-System: 0.12 (ns), 0.033 (*), 0.002 (**), < 0.001 (***).

## Supplementary Information

Supplementary Legend.

Supplementary Figure S1.

## Data Availability

All raw data and processed images generated and analysed during the current study are available from the corresponding author on reasonable request.

## Abbreviations

2D: Two-dimensional
3D: Three-dimensional
a-CaP: Amorphous calcium phosphate
ASCs: Adipose tissue-derived mesenchymal stem cells
BMP-2: Bone morphologic 2
BTE: Bone tissue engineering
CAM: Chorioallantoic membrane
COL: Collagen
ECM: Extracellular matrix
GAG: Glycosaminoglycan
H&E: Haematoxylin and eosin
HA: Hyaluronic acid
HIF-1α: Hypoxia-inducable factor 1α
ID: Incubation day
microCT: Microcomputed tomography
MRI: Magnetic resonance imaging
MSCs: Mesenchymal stem cells
MTs: Microtissues
NaCl: Sodium chloride
PBS: Phosphate buffered saline
PLGA: Poly-lactic-co-glycolic acid
ROI: Region of interest
SCs: Single cells
SFM: Serum-free media
TE: Tissue engineered
TEC: Tissue engineered construct
