# Supplementary material for: 3D-microtissue derived secretome as a cell-free approach for enhanced mineralization of scaffolds in the chorioallantoic membrane model
Source: Sci Rep. 2021 Mar 8;11:5418. doi: 10.1038/s41598-021-84123-x (PMC7940489; doi:10.1038/s41598-021-84123-x)
Supplement: Supplementary file 1 — Supplementary Legend. [file 41598_2021_84123_MOESM1_ESM.docx]

**Figure S1. Representative histological images corresponding to the time course (Figure 2**). H&E staining shows cell infiltration (A) and new vessel formation (B) on day 1. On day 3, collagen (C) and glycosaminoglycan (D) are detectable. Von Kossa staining detects calcium deposits in the surface (E) and shows automatic detection of staining intensity using Image J software. Images A-D show a section of the interphase. Scale bar 100 um.
