# Supplementary material for: 3D-microtissue derived secretome as a cell-free approach for enhanced mineralization of scaffolds in the chorioallantoic membrane model
Source: Sci Rep. 2021 Mar 8;11:5418. doi: 10.1038/s41598-021-84123-x (PMC7940489; doi:10.1038/s41598-021-84123-x)
Supplement: Supplementary file 2 — Supplementary Figure S1. [file 41598_2021_84123_MOESM2_ESM.docx]

**3D-microtissue derived secretome as a cell-free approach for enhanced mineralization of scaffolds in the chorioallantoic membrane model**

Lukas Otto^1,2*^, Petra Wolint^1,2*^, Annina Bopp^3,4^, Anna Woloszyk^3,5^, Anton S. Becker^6^, Andreas Boss^6^, Roland Böni^7^, Maurizio Calcagni^1,2^, Pietro Giovanoli^1,2^, Simon P. Hoerstrup^3,8^, Maximilian Y. Emmert^3,8,9,10**^ and Johanna Buschmann^1,2**^

^1^Division of Surgical Research, University Hospital of Zurich, Zurich, Switzerland

^2^Plastic Surgery and Hand Surgery, University Hospital Zurich, Zurich, Switzerland

^3^Institute for Regenerative Medicine, University of Zurich, Zurich, Switzerland

^4^Hospital Limmattal, Schlieren, Switzerland

^5^Department of Orthopaedic Surgery, University of Texas Health Science Center San Antonio, San Antonio, TX, USA

^6^Institute for Diagnostic and Interventional Radiology, University Hospital of Zurich, Zurich, Switzerland

^7^White House Center for Liposuction, Zurich, Switzerland

^8^Wyss Translational Center Zurich, University of Zurich & ETH Zurich, Zurich, Switzerland

^9^Department of Cardiovascular Surgery, Charité Universitätsmedizin Berlin, Berlin, Germany

^10^Department of Cardiothoracic and Vascular Surgery, German Heart Center Berlin, Berlin, Germany

* Shared first authorship

** Shared last authorship


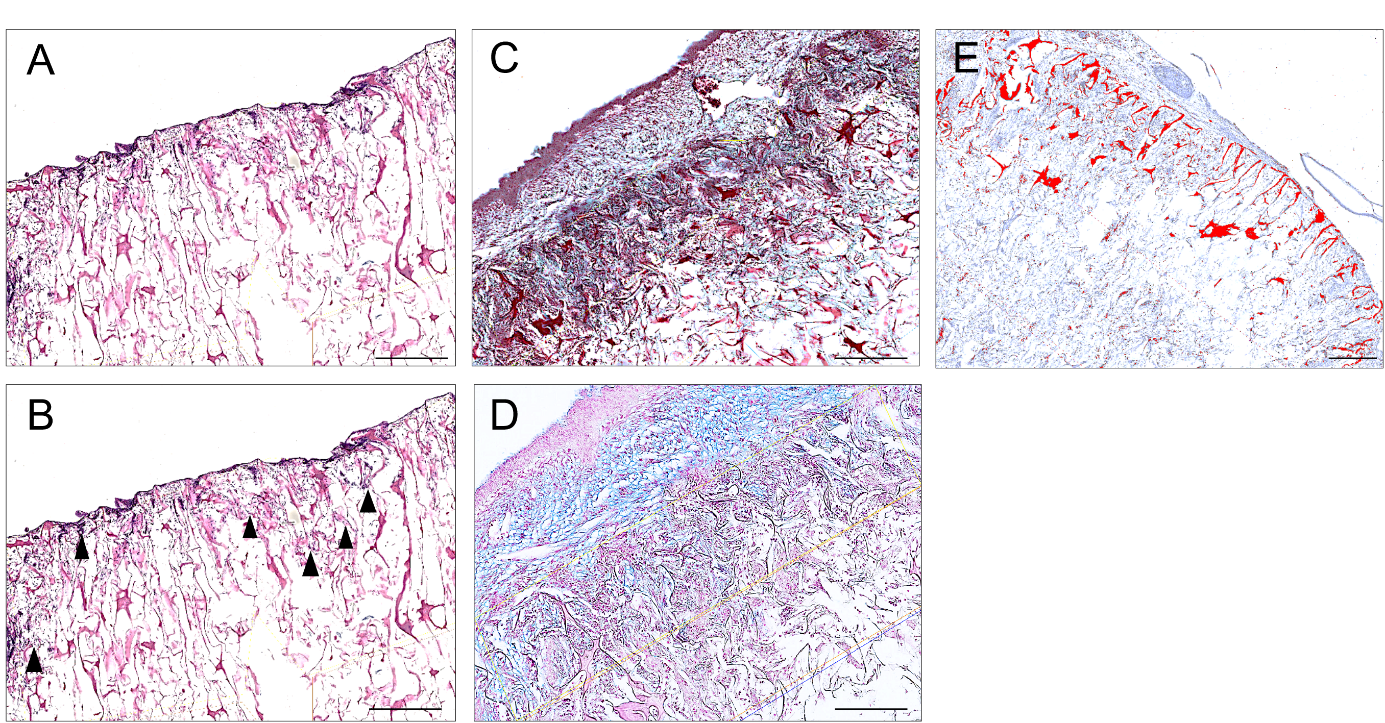


Figure SI 1
